# Inter-atrial septum dissection during atrial fibrillation ablation via atrial septal defect: A rare complication

**DOI:** 10.1016/j.hroo.2026.02.001

**Published:** 2026-02-09

**Authors:** Chizute Ogbedeh, Cinzia Monaco, Ciro Ascione, Pierre Jaïs, Nicolas Derval, Mélèze Hocini

**Affiliations:** 1 School of Clinical Medicine, University of Cambridge, Cambridge, United Kingdom; 2 Cardiology Hospital Haut-Lévêque, CHU Bordeaux, Pessac, France; 3 IHU LIRYC (Cardiac Electrophysiology and Modelling), CRCTB (Inserm u1045), University of Bordeaux, Bordeaux, France

**Keywords:** Atrial fibrillation, Catheter ablation, Marshall-plan, Peri-procedural complications, Atrial septal defect, Peri-cardial effusion, Transseptal puncture

## Abstract

Catheter ablation has become an established treatment modality for atrial fibrillation, particularly in patients with refractory or intolerant responses to medical therapy. The Marshall-Plan has garnered interest in this field for its ability to achieve durable posterior mitral isthmus ablation. This anatomically-based approach combines alcohol ablation of the vein of Marshall with pulmonary vein isolation, mitral roof, and isthmus ablation, and a cavotricuspid isthmus line. Despite its effectiveness, procedural complications, although rare, must be considered.

Here, we report a case of suspected inter-atrial septum dissection during Marshall-Plan atrial fibrillation ablation, complicated by the development of a saline transudative peri-cardial effusion. The study adheres to the Declaration of Helsinki.

**What we learned**

▪ Consider inter-atrial septum dissection as a potential differential for tamponade during atrial fibrillation catheter ablation, particularly when transseptal puncture is difficult.
▪ Intracardiac echocardiography, fluoroscopy with contrast or trans-esophageal echocardiography may provide valuable insight into the mechanism of peri-cardial effusion post-ablation.
▪ Catheter stability during ablation of the left atrium is best achieved through postero-inferior puncture of the inter-atrial septum—using a persistent foramen ovale or atrial septal defect to gain access into the left atrium may compromise stability.

## Introduction

**Figure 1:**
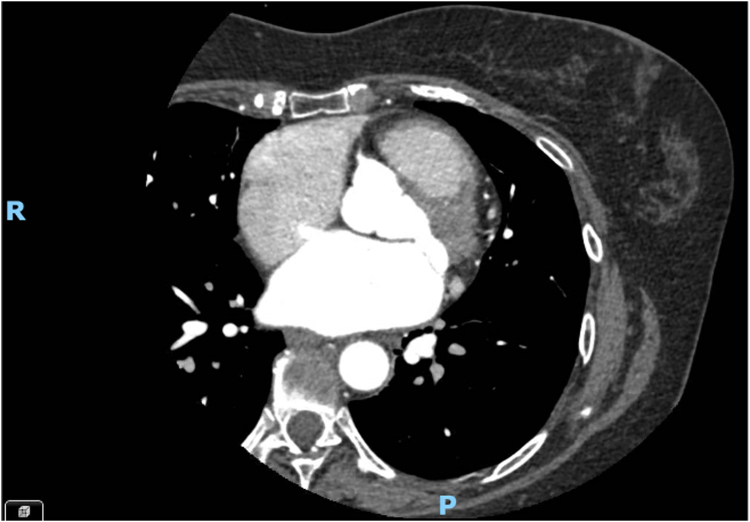
Pre-operative computed tomography scan of atrial septal defect.

A 60-year-old woman with persistent atrial fibrillation (AF) was admitted for catheter ablation following unsuccessful electrical cardioversion (3 shocks at 360 J). The pre-procedural electrocardiogram demonstrated atypical atrial flutter (66 bpm) with a narrow QRS complex. A pre-operative computed tomography scan identified a small atrial septal defect (ASD), measuring 10 x 3 mm, with no evidence of thrombus (Figure 1).

## Methods and Results

**Figure 2:**
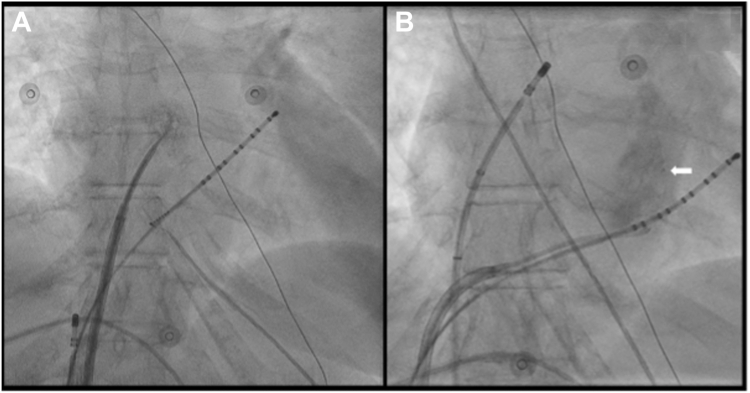
Fluoroscopy-guided transseptal puncture **(A)**, Ethanol infusion into the Vein of Marshall **(B)**. *White arrow* = Vein of Marshall post-ethanol ablation.

**Figure 3:**
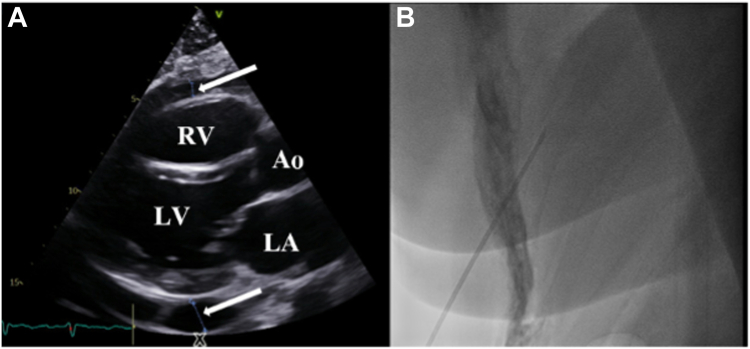
Pericardial effusion **(A)**. Subxiphoid puncture for pericardial drainage **(B)**. Ao = aorta; LA = left atrium; LV = left ventricle; RV = right ventricle; *White arrow* = pericardial effusion.

The procedure was conducted under conscious sedation. 8000 IU of heparin were administered, with an additional 4000 IU given during the procedure. The coronary sinus catheter was placed early; initial fluoroscopy-guided transseptal puncture (TSP) proved challenging (Figure 2A) because of anatomical difficulty; therefore, further attempts were abandoned. As a result, the pre-existing ASD was used to gain access to the left atrium (LA). However, the ablation catheter (THERMOCOOL SMARTTOUCH®, Biosense Webster) was difficult to maneuver into the LA, which necessitated repositioning. Subsequent mapping was followed by ethanol ablation of the vein of Marshall (Figure 2B) which was achieved without incident. Mapping was performed using the OCTARAY™ multielectrode catheter (Biosense Webster). Following successful isolation of the left pulmonary veins, difficulties arose during ablation of the septal aspect of the right pulmonary veins, with repeated catheter slippage into the right atrium, requiring repositioning into the LA. The catheter was intermittently unstable in the LA for about 10 minutes while attempts were made to achieve sufficient contact for ablation. During these exit and re-entry maneuvers, the patient’s arterial pressure dropped to 60/40 mm Hg. This was noted 30 minutes after ethanol infusion into the vein of Marshall and 10 minutes after use of the ASD for access into the LA. Subsequent echocardiography identified a 1.6 cm peri-cardial effusion as the cause of cardiac tamponade (Figure 3A). Protamine (40 mg) was administered to reverse the anti-coagulation, and immediate peri-cardial drainage was performed via subxiphoid puncture (Figure 3B). A total of 250 mL of lightly blood-stained, serous fluid was aspirated and sent for biochemical analysis. There was no evidence of bright red or pulsatile flow, suggesting the bleed was of low pressure and consistent with venous oozing or fluid seepage. Analysis characterized the effusion to be transudative in nature (protein = 3.1 g/L, LDH >80 IU/L). The following day, an echocardiogram confirmed resolution of the effusion.

## Discussion

Marshall-Plan AF ablation,^1^ while effective, carries a risk of complications. Cardiac tamponade or effusion is a rare occurrence after catheter ablation but is second only to vascular complications post-procedure.^2^ 2 differential diagnoses were considered to explain the pericardial effusion: (1) a hypersensitivity reaction to ethanol infusion, or (2) an iatrogenic interatrial septum dissection caused by catheter manipulation with irrigation of the pericardium by the ablation catheter. The transudative nature of the fluid supports the second hypothesis. Difficulties encountered during ablation near the septal region of the right pulmonary veins suggests insufficient support from the ASD route; these mechanical instabilities likely contributed to the septal injury. The likely mechanism of injury involves dissection of the septum secundum with subsequent entry into the extracardiac space, ablation catheter irrigation, and local bleeding.^3^ This would have been best confirmed by intracardiac echocardiography; however, it was not available during the procedure. Diagnosis by fluoroscopy with contrast injection or trans-esophageal echocardiography were alternative options,^3^ but were not performed in this study, limiting our ability to be certain. Using a persistent foramen ovale (PFO) or ASD for left atrial access during AF ablation has been associated with procedural outcomes similar to those achieved with TSP, particularly in interventions such as left atrial appendage closure.^4^ However, for AF ablation, where stability is crucial for successful ablation of the posterior aspect of the LA, a postero-inferior septal puncture is preferable^5^ to ensure catheter stability and minimize the risk of iatrogenic complications. This case highlights the potential for catheter instability and complications when using a central or anterior septal access point in AF ablation. Lack of intracardiac echocardiography imaging during this procedure limited our capacity to fully characterize the mechanism of dissection. The value of real-time septal visualization in this case would have prevented complications of this nature.^6^

## Conclusion

Although using a PFO or ASD for left atrial access may initially seem safer by avoiding the challenge of TSP, it may not provide the optimal pathway for AF ablation, where a more posterior access facilitates catheter stability.

## Disclosures

Nicolas Derval received modest consulting fees and speaking honoraria from Biosense Webster. Nicolas Derval and Pierre Jaïs received modest speaking honoraria from Boston Scientific. All other authors have reported that they have no relationship relevant to the contents of this paper to disclose.
